# Orthodontic Treatment Based on Wearable Mirror-Type Oral Prosthetic Tongue Flap without Bracket Correction

**DOI:** 10.1155/2021/4979681

**Published:** 2021-06-10

**Authors:** Yagang Chen, Xiaoxiao Wu, Wenwen Wu

**Affiliations:** The Affiliated Hospital of Xuzhou Medical University, Xuzhou 221000, Jiangsu, China

## Abstract

White teeth can make people full of confidence and satisfy the concept of modern life from the love of beauty. Due to the fusion of computer-aided design and teeth, invisible orthodontics has become the focus of research. Invisible orthodontic treatment technology can predict the results of orthodontics. How to automatically calculate the position and posture of the teeth in the middle stage of orthodontics is the key point of the treatment technology. In order to solve this problem, this article is divided into two parts to start research. Aiming at the problem of tooth orthodontic path planning, quaternion is used to define the tooth posture, combined with the initial posture and target posture of the tooth. A two-stage method is given to plan a collision-free path for the orthodontic tooth. In the first stage, the quaternion spherical linear interpolation and position linear interpolation are used to obtain the intermediate posture of the tooth during orthodontics, and the initial value of the orthodontic stage is obtained, and the obtained intermediate posture is used as a sampling node to apply to the next stage. In the second phase, considering the problem of orthodontic collision and interference, a scheme for calculating the priority of orthodontics is proposed, and the random node expansion part in the RRT (Rapid-exploration Random Tree) algorithm is improved. The initial value of the orthodontic phase is used to calculate the initial value of the iteration. Finally, a path with no collision and the least number of orthodontic stages is searched from the random tree of each tooth node. The experimental results and analysis show that this method can quickly and effectively solve the orthodontic path of teeth, and it is used clinically. The clasp-free invisible correction technology pushes the molars far away to leave gaps for treating patients with mild to moderate overcrowding. The treatment time should be reduced by at least 30%; the stability of the gaps and the long-term healing effect of the treatment provide a reference.

## 1. Introduction

In the past few years, the number of dental patients has remained high. The most common dental problem is pain due to tooth deformity. The normal treatment method is deformity correction. The traditional treatment method is no longer satisfactory. For people's beautiful image, wearable mirror-type oral restoration technology is a new type of clinical orthodontic treatment with good concealment and aesthetics and has little impact on the normal life of patients. Most dysfunctional patients have long teeth. Waist circumference is a common clinical manifestation of dysfunction. According to different populations, the number of prostheses is divided into light, medium, and heavy. Clinically, according to the degree of congestion, the method of relieving congestion is also different. For mild congestion and/or soft tissue profile that does not need to be improved, nonextraction treatment can be used to push the molars to the middle and/or expand the arch and film. Methods such as cutting can relieve congestion, and for severe congestion and/or large soft tissue profile protrusion, it is often necessary to remove the congestion and/or improve the soft tissue profile through tooth extraction. For some cases of mild congestion combined with protruding soft tissue profile or moderate congestion, between extraction and nonextraction, some nonextraction orthodontic measures can also be used to relieve congestion and improve soft tissue profile. One of the most common ways is to push the molars to move distally to increase the gap, so as to relieve crowding and/or improve the profile.

Qin Hongjin believes that children should use audio suppressors when practicing oral English and has developed a new type of language suppressor specifically for children's oral practice. Its structure includes a tongue depressor body and a separate structure for protecting the tongue depressor cup. The main material is a colored plastic plate, the front end of which is at the front end, and the thickness of both ends is gradually reduced to form an arc shape and lightly touch the surface of the silicone protective cover. The voice suppressor is reasonably designed, easy to use, and safe. After use, it can promote children's clinical work and oral exercise training, but the high production cost will increase the burden on the patient and is not suitable for actual situations [1]. Lan Li analyzed the clinical effects of unsupported invisible correction technology in orthodontics. The control group used conventional fixation devices, and the observation group used invisible correction technology without stents. The satisfaction and treatment effects of the two groups were compared and analyzed. To conclude (*P* < 0.05), the orthodontic and stentless infiltration technique has a good corrective effect on orthodontic patients and can improve patient satisfaction. However, the data is not comprehensive, and more data are needed for experiments to get accurate results [2]. Zhiqiang studied the importance of orthodontic use without square brackets. The method divided 62 orthodontic patients in our hospital into two groups according to the double-blind method from December 2018 to December 2019. The conventional group (traditional fixed treatment technology) had 31 cases, with 31 cases in the test group (without stent technology). And, the clinical efficacy of the two patient groups was compared. The results showed that the total clinical efficacy rate of the test group was 96.77%, and aesthetic comfort (8.31 ± 0.89), stable position function (9.76 ± 0.22), chewing function (8.75 ± 1.19), convenience (9.61 ± 0.29), tongue function (8.98 ± 0.23), midline (0.14 ± 0.02), coverage (0.12 ± 0.06), posterior teeth (3.93 ± 0.19), and anterior teeth (0.52.0.06) were higher than those in the conventional group, and the difference was statistically significant (*P* < 0.05). In conclusion, the benefits of unsupported orthodontic technology are obvious, and it can effectively improve patient satisfaction and tooth arrangement. But he did not consider the special situation of actual patients [3, 4].

By understanding the process of invisible orthodontics, it can be known that during the invisible orthodontic treatment the dental technicians use the virtual orthodontic system to design the master model of the appliance at each stage to provide data for the subsequent processing of the appliance. The key point is how to design each appliance. Stage of the master model of the appliance is similar to traditional appliances, invisible appliances at a certain stage also need to provide a certain amount of orthodontic force to a misaligned tooth to translate or rotate it in the target direction, so that the misaligned tooth can reach the target position of the stage Overall, the current invisible correction technology is based on technology related to computer-aided design, including separating teeth and gum models, restoring tooth models, simulating tooth movement, and formulating procedures to correct the orthodontic stage [5, 6]. This article will focus on the problem of orthodontic path planning in invisible correction, which can assist orthodontists in designing correction schemes, and reduce labor when designing orthodontic models for dental arrangement technicians. Therefore, the development of efficient related algorithms has important application value. At the same time, it is also the key and difficult point in invisible correction technology.

## 2. Orthodontic Method of Orthodontic Correction Technology without Brackets

Orthodontics refers to the method of correcting teeth to remove deformities and deformities. Orthodontists mainly study the causes and mechanisms of deformities, analyze and diagnose them, and then do a good job of treatment and prevention of deformities [7]. This treatment method mainly uses various orthodontic devices to relax the obstruction and adjust the coordination between the entire tooth and the jaw, including the relationship between the upper and lower jaws, the relationship between the upper and lower teeth, and the relationship between the teeth and the lower jaw. The relationship finally achieves the stability, balance, and aesthetics of the oral system and the mandible The device can be worn outside or through the mouth to apply appropriate “biological force” on the teeth, thereby affecting the alveolar and jawbone and causing physiological changes so that the teeth can move to correct rejection. According to the needs of different doctors and patients for effectiveness, aesthetics, and comfort, more treatment options can be provided, such as movable stents, fixed laboratory stents, self-locking arms, ceramic stents, invisible armless stents, and invisible tongue stents. The main feature of the functional arm is that the corrective ability of orthodontic teeth mainly comes from the muscular strength of the patient's mouth and jaw or the constant and soft corrective force [8, 9]. Most functional supports are movable supports, such as two-piece supports, and Frankel, but some functional supports are fixed supports, such as Forsus and Herbst.

### 2.1. Fabrication of Lingual Brackets

**Figure 1:**
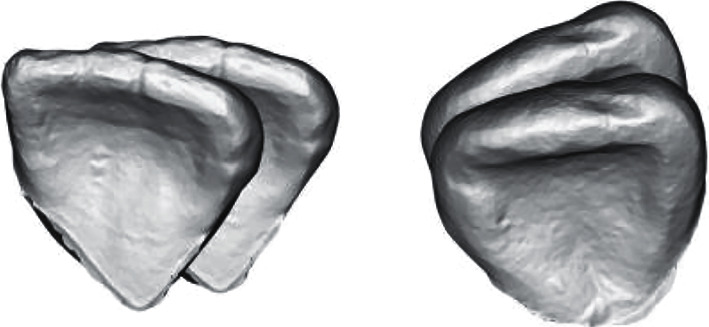
The initial and target positions of the central incisor (the picture is taken from https://image.baidu.com/).

In the medical field, especially in the field of dentistry, the most common manufacturing method for personalized precision metal products is investment casting. The investment casting method of dental restorations, personalized implants, and other medical devices is first to make the wax model of the product, use plaster embedding powder to embed the wax model into a mold, and then bake and cast at high temperature. Mold, evaporate the wax mold to form a cavity, and then use centrifugal casting or vacuum casting to fill the mold. Individualized orthodontic brackets cannot be manufactured using standard bracket machining methods due to their small size and high precision requirements, and can only be manufactured by investment casting [10, 11]. The process flow of investment casting is usually pressure molding, wax mold pressing, wax mold assembly, dipping coating, sanding, hardening and drying, dewaxing, roasting, pouring, sand falling, and cleaning. Due to the many process links and the complicated process, the quality of the final casting is greatly affected by many factors and is not easy to control. In terms of making wax molds, the traditional method is hand-made by technicians. This method has a long processing cycle and cumbersome steps, as shown in Figure 1.

The emergence and application of rapid prototyping technology using wax as a material has greatly improved the efficiency and precision of wax molding. However, with the investment casting method, the wax mold and the mold are disposable and cannot be reused. The cost of producing individual parts in small batches is high. Similarly, the investment casting process cannot solve the problem of forming a single part with an internal cavity structure. These limitations of investment casting make the manufacture of lingual brackets very difficult [12, 13]. Therefore, it is very important to find a suitable manufacturing method to shape the lingual bracket.

### 2.2. Selective Laser Melting Technology

Additive manufacturing technology (also called rapid prototyping) is a high-tech technology that uses the material accumulation method developed in the mid-1980s to manufacture natural products. This technology combines computer-aided design (CAD) and computer-aided design through computer, laser, precise transmission, and numerical control technology for assisted manufacturing (CAM) integrated together, and in a short time through the layer-by-layer construction method to produce instant information. Product samples that do not use traditional processing equipment and company technology, product development extend the cycle, and improve the company's competitiveness. Compared with traditional mechanical manufacturing methods, additive manufacturing technology can perform rapid prototyping under any complex structure. In the manufacturing process of one-off or small batch production, it has the advantages of low manufacturing cost and short cycle [14, 15]. Therefore, it is widely used in the engineering industry. Additive manufacturing technology is a pioneering material casting technology. It uses the principle of discrete/stacked casting to freely determine the path, constraint conditions, and methods for retaining the accumulation, and forms a three-dimensional unit through material accumulation and covering.

## 3. Experiments on Orthodontics without Brackets

### 3.1. Orthodontic Research Indicators

The overall effective treatment rate is divided into extremely effective, effective, and ineffective. Extremely effective: after treatment, the patient's teeth are clean, the coverage rate is accurate, and it can be eaten normally without worry. Effective: the patient's tooth failure is corrected and the layout is relatively neat. The coverage rate is accurate. Ineffective: the treatment does not meet the above criteria, the dental dysfunction is obvious, and there is no sign of improvement. The overall effect rate = (extremely effective + effective)/total number of cases × 100% [16, 17]. The direct calculation method of the arithmetic mean of the small sample: it is suitable for the data with the sample number of less than 30 cases (called the small sample). The arithmetic mean can be obtained by adding up the values of the variables and dividing it by the number of cases:

(1) $$\begin{array}{c} G={\mathrm{lg}}^{-1}\left(\frac{\sum \mathrm{lg}x}{n}\right). \end{array}$$

Large sample arithmetic mean simple calculation method: when there are many variables, it is more troublesome to use the above direct method, and simple calculation method can be used.

(2) $$\begin{array}{c} \frac{3+x3-xn}{n}=\frac{\sum x}{n}. \end{array}$$

(1) Direct calculation method: when the number of variables is small and the value is small, the direct calculation method can be used.
  (3) $$\begin{array}{c} \bar{x}=x_{0}\frac{\sum f\;d}{n}\left(i\right). \end{array}$$
In the formula, x is the value of each variable of the sample; for example, the geometric mean of 2, 4, and 8 is as follows: *G* = (2 × 4 × 8)1/3 = 4. The arithmetic mean is as follows: (2 + 4+ 8)/3 = 4.67, which is larger than the geometric mean [18, 19].
(2) Logarithmic calculation method: when the number of variables is more than 3 and the value is large, the logarithmic calculation method can be used.
  (4) $$\begin{array}{c} Sp=3C\sqrt{\frac{PQ}{n}}. \end{array}$$
(3) Frequency calculation method: when the number of variables is large and grouping is required, the frequency calculation method is used.
  (5) $$\begin{array}{c} G\;=\;\mathrm{lg}∠10\left(\sum \mathrm{lg}x\right). \end{array}$$
(4) Weighted calculation method: when the number of variables is large, the logarithmic calculation method is more troublesome, and the weighted calculation method can be used.
  (6) $$\begin{array}{c} G:\mathrm{lg}-l\left(\sum gx\right). \end{array}$$

### 3.2. Sagittal Control

In the invisible orthodontic technique without brackets, in the sagittal movement of the teeth, the highest predicted success rate is the lateral incisor of the upper jaw, while the central incisor of the canine and jaw has the lowest predicted success rate [20, 21]. For dog teeth over long distances, both traditional fixed orthodontic techniques and invisible nonstented orthodontic techniques can cause the dog's teeth to move sideways. However, the armless invisible orthodontic technology is a complete device with a crown wrap and a center of gravity and uses fixed correction technology to reduce the inclination of the dog. Invisible orthodontic bracket technology can also effectively move the molars remotely to increase space, reduce the number of molars, and adjust the occlusal relationship of the molars, provided that the anchoring can be slightly reduced.

Frequency calculation method: when there are a large number of variables and grouping is required, frequency calculation method is used [22, 23]:

(7) $$\begin{array}{c} \frac{1}{m}=L+\frac{I}{\sum n+s}\left(\frac{n}{2}-C\right), \end{array}$$

where *L* is the lower limit of the group where the median is, the frequency of the group where the median is added is the group distance, Ru is the total number of cases, and *C* is the cumulative frequency before the group where the median is.

The two-sample *t*-test is the most basic method in statistics, which is used to infer whether the respective population means represented by two independent samples are equal. The statistical calculation formula is as follows:

(8) $$\begin{array}{c} S=x1-t\sqrt{S_{c}^{2}}\left(1/n_{1}+1/n_{2}\right). \end{array}$$

The standard deviation is used to calculate the coefficient of variation. When the two sets of observations (that is, variables) have different units, or when the two means differ greatly, the standard deviation cannot be used to directly compare the degree of dispersion. In this case, the coefficient of variation (CV) should be used as in [24, 25]. The smaller the coefficient of variation, the smaller the degree of dispersion of the observed values; on the contrary, the larger the degree of dispersion of the observed values:

(9) $$\begin{array}{c} \text{CV}:S+\sqrt{\frac{n1}{n2}}-\left(n1^{2}+n2^{2}\right). \end{array}$$

The standard deviation is used to calculate the standard error of the mean, and finally a t-test is performed to determine the statistical significance:

(10) $$\begin{array}{c} S_{\bar{x}}=\frac{n2-1}{t\sqrt{n^{2}}}. \end{array}$$

The standard error of the rate of use is calculated as the standard error of the rate (sp) and the degree of reliability of the response rate [26, 27].

(11) $$\begin{array}{c} S_{1/X}=\text{sp}+\sqrt{\frac{S}{n_{2}+n1_{t-1}}}+b. \end{array}$$

For data of the same nature that represents the size of the sampling error, the smaller the standard error, the smaller the sampling error, that is, the higher the reliability of inferring the population from the sample [28, 29].

(12) $$\begin{array}{c} S^{2}=\sqrt{b\frac{\text{PQ}}{n}}. \end{array}$$

Enter the data of this study into the statistical software SPSS21.0, and the measurement data is expressed as x ± s, using *t*-test [30]. The count data is expressed as %, using the *χ*2 test; if *P* < 0.05, the difference is statistically significant.

## 4. Application Effect of Bracketless Invisible Correction Technology in Orthodontics

### 4.1. Comparison of Patient Treatment Effects

70 patients receiving orthodontic treatment were randomly selected as research subjects. Using the coin toss method, the patients were randomly divided into an observation group (treatment with invisible braces and treatment with invisible braces) and a control group (treatment with conventional fixation devices), with 35 cases in each group. All patients agreed to participate in this study. The diagnostic criteria were mild to moderate obstruction and the first maxilla of the patient's maxilla was locked in the middle mouth of the first permanent mandible. This study excluded patients with obvious dental problems, periodontal disease, opening type, abnormal opening angle, or low level of cooperation. The treatment effect of the two groups of patients was compared. The time was significantly shorter than the control group; at *P* < 0.05, the difference was statistically significant.  Table 1 provides the comparison of treatment effects between the two groups of patients

Patients in the control group were treated with traditional treatment methods. After the patient is hospitalized, the doctor uses X-rays to understand the patient's oral condition and treats the patient with metal clips and straight wire instruments, which are glued to the patient's mandibular denture. The patient should resume conventional treatment and change the use of arch cables. The sequence of arc changes is thin and thick, round, and square. Stainless steel is made of titanium and nickel. During the treatment, the patients were taught oral hygiene and instructed to keep the oral cavity clean and healthy, as shown in Table 2.

The quality of the results of automatic tooth arrangement can be tested by comparing the results of manual tooth arrangement, and computer-aided design can provide accurate measurement methods. The comparison method of dental jaw measurement is mentioned in the cited literature, the measurement items are shown in Table 2, and the sample data are used as the experimental data to carry out automatic and artificial tooth arrangement results according to the measurement of jaw width, full jaw, and upper and lower jaw coverage, respectively.

**Figure 2:**
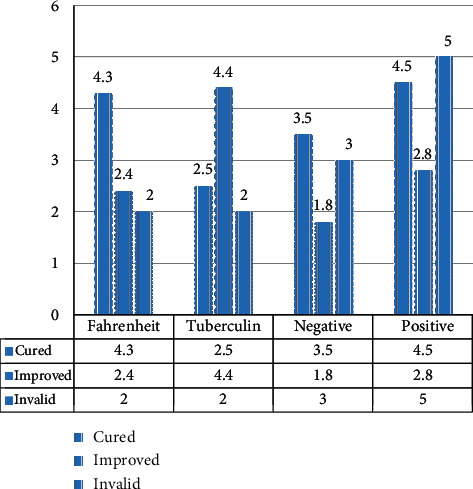
The results of treating a disease.

As shown in Figure 2, the count data is the record obtained by counting the number. For example, the Fahrenheit reaction and tuberculin test only record the number of positive and negative reactions; with regards to the results of the treatment of the disease, it records the number of people who are all cured, improved, and invalid. The standard lingual bracket adopts a prefabricated bottom plate, which does not fit the tooth surface. And the bottom plate is relatively small. This is because an excessively large bottom plate will cause the bottom plate and the tooth surface to require more adhesive when bonding, so the standard brackets that are generally designed are easy to fall off in clinical applications.

**Figure 3:**
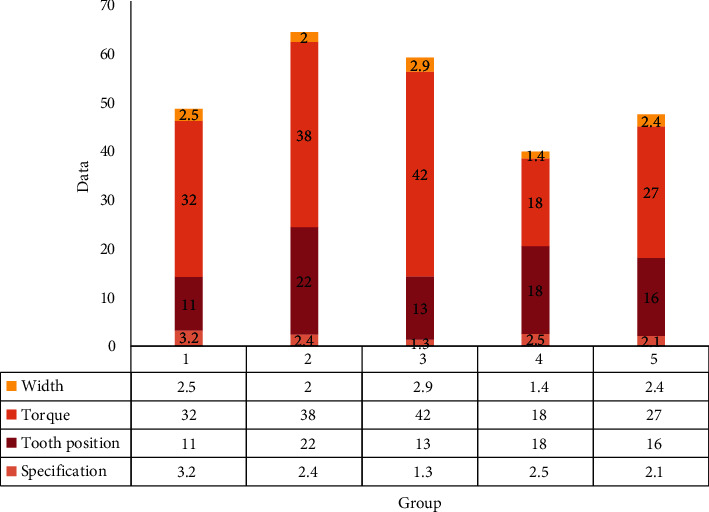
Lingual bracket parameter table.

It can be seen from Figure 3 that *c* is the thickness of the groove wall, *P* is the force exerted by the archwire, *b* is the height of the archwire in the groove, *I* is the length of the groove, and SA is the unit yield strength of the material. The greater the height of the archwire in the groove, the greater the force *P*, and the thickness of the groove wall should be increased; the longer the length of the groove, the higher the strength of the material, and the thickness of the groove wall can be reduced accordingly. Although the miniaturization of brackets is a direction for the development of orthodontic brackets, it is impossible to reduce the volume of the brackets unlimitedly.

**Figure 4:**
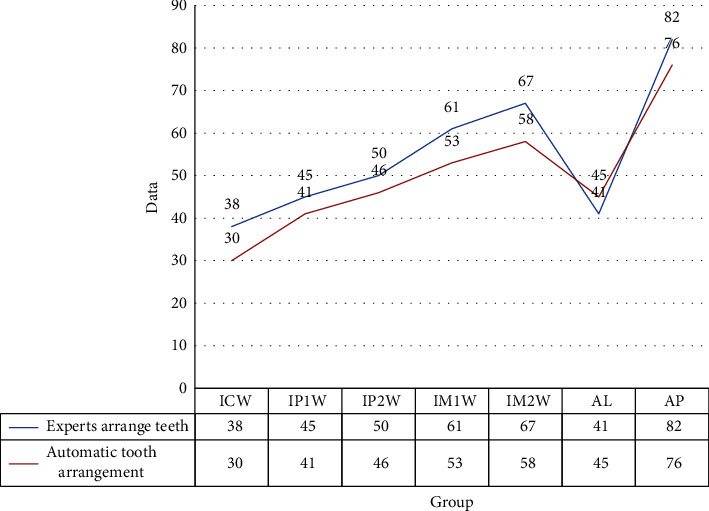
Measurements of the upper jaw.

Figure 4 shows that the result of automatic tooth arrangement is smaller than that of manual tooth arrangement, which proves that the algorithm in this paper can replace artificial tooth arrangement. Among them, when measuring the jaw width, the result of artificial tooth arrangement is slightly wider than the result of automatic tooth arrangement, and the difference is within 1 mm. The average difference in the measurements of jaw width is not significant. On the AP measurement of jaw circumference, artificial arrangement of the upper jaw is 1.16% longer than the automatic arrangement, and the lower jaw is 0.12% shorter. It can be seen that the tooth model of the automatic arrangement is closer.

### 4.2. Comparison of Language Function Scores

The SPD, PLI, SBI, and GI of the observation group after treatment were significantly higher than those before treatment (*P* < 0.05), while the PLI, SBI, and GI of the control group were significantly higher than before treatment (*P* < 0.05). Although the scores of GI and GI were higher than those of the control group, the difference was not statistically significant (*P* < 0.05), as shown in Table 3.

After the observation group was hospitalized, the doctor performed a special check on the patient to ensure that invisible orthodontics can be treated without a bracket, and then a lot of preparatory work was done. Details are as follows. The relationship between the upper and lower occipital bones of the patient's mouth is determined by the silicone model. Patient's dental film and diagnosis and treatment records, and an X-ray of the skull. The patient's oral disease was analyzed in detail and accurately and fully communicated with the patient before treatment to understand the patient's willingness to treat. The invisible orthopedic center of our hospital corrects patients. The physician is responsible for the task of binding the ligaments and applying the correction to the patient. The frequency of rectal replacement of the patient is once every two weeks. There are 4 pairs of orthoses during the first treatment. The patient will return to the hospital every two months. Daily corrective wear should be ≥22 hours. The doctor should adjust the correction plan according to the patient's specific correction status at the time of diagnosis, formulate a complete correction plan for the patient, and complete the next treatment with an appropriate treatment method. The total calibration time range should be 5 months to 1.5 years.

The postoperative comfort, aesthetics, portability, and function scores of the observation group were better than those of the control group, and the difference between the two groups was statistically significant (*P* < 0.05). The criteria for evaluating the effect of treatment are as follows: the dysfunction of patients with prosthetic dysfunction is effectively corrected, the prosthesis is evenly arranged, there is almost no covering between the teeth, and the molar relationship is neutral or removed; if the patient's oral condition and the following criteria are not met, the treatment is invalid. The actual treatment rate is calculated based on the number of effective patients, and then the patient classification method is used to classify the postoperative comfort, aesthetics, portability, and speech function. The score ranges from 0 to 10, with 0 being very unsatisfactory and 10 being very satisfied. Patients can score according to their own situation. The details are shown in Table 4.

**Figure 5:**
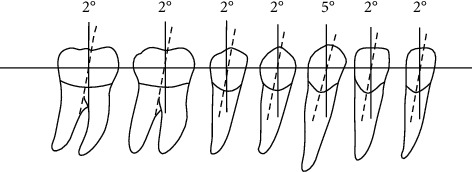
Average mandibular crown inclination at optimal jaw.

Proximal and distal inclination of teeth (coronal angle, axial inclination): the angle formed by the long axis of the clinical crown of the tooth and the perpendicular to the jaw plane is the crown or axial inclination, which represents the inclination of the proximal and distal teeth. The coronal angle is positive when the gingival end of the long axis of the clinical crown is inclined distally, and the coronal angle is negative when inclined to the mesial. The crown angles of normal jaws are mostly positive, and the number of crown inclination angles of each tooth type is similar as shown in Figure 5.

It can be seen from Figure 5 that the central oral cavity edge of the first permanent molecular molar is sealed in the central oral cavity of the first permanent molecular jaw, and the peripheral ridge of the first permanent molar does not include the central edge of the second permanent molar of the jaw. The tip of the middle tongue is hidden in the central fossa of the second permanent molar of the lower jaw, and the mouth end of the upper jaw is closed in the adjacent area of the opposite lower jaw. Mitral valve of the lower jaw: the upper jaw is closed between the lower jaw and the first mitral valve, the pin is slightly close to the center, the upper jaw covers the lower incisors, and the upper and lower arches are the same.

**Figure 6:**
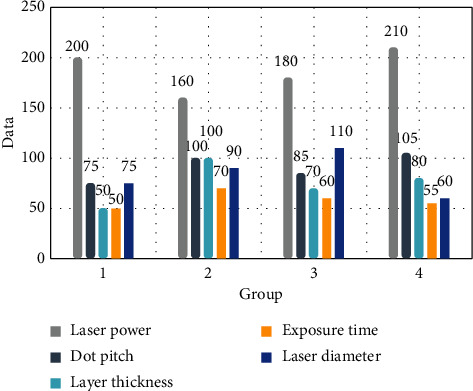
SLM print manufacturing parameters.

As shown in Figure 6, the SLM print manufacturing machine has good sealing performance and the presence of protective gas makes the oxygen content in the manufacturing environment less than 0.01%. This machine can be used to print 316 stainless steel, cobalt-chromium alloy, and titanium alloy and it also has a good printing effect for titanium alloys with relatively high activity. For different printing parameters, the effect of printed parts is very different. The important parameters are laser power, scanning speed, dot spacing, and so on. These parameters are obtained by RENISHAW after a large number of experiments and have a high effect on SLM processing parts and reference value.

**Figure 7:**
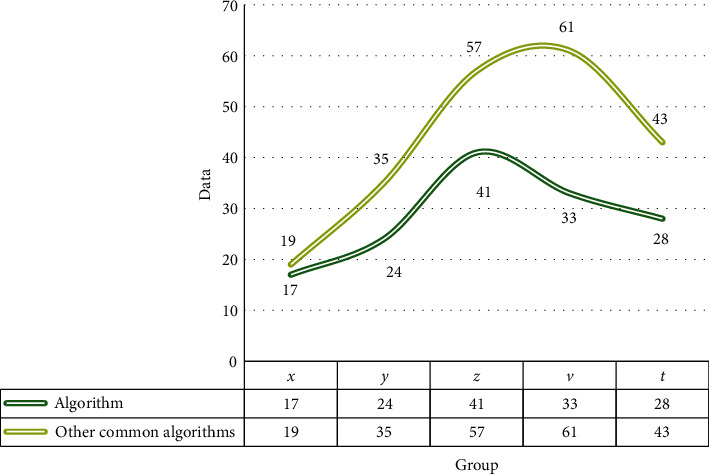
Comparison of total translation and rotation movement.

As shown in Figure 7, in order to realize the application of low artificial teeth with computer-aided design in the field of orthodontics, a method for automatically adjusting misaligned teeth based on tooth position optimization has been developed. Experiments show that prosthesis can work and move, and rotating the prosthesis to exclude it from the target site, the result of the prosthesis obtained, which can significantly reduce the potential of the human prosthesis. Using the existing tooth placement algorithm, the calculation efficiency becomes more efficient. The lower the coming, the clinical dental treatment has obvious advantages in terms of the cost of moving teeth.

**Figure 8:**
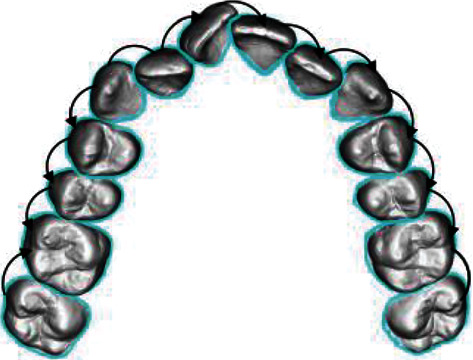
Orthodontic priority of sample dental data.

As shown in Figure 8, from the displayed intermediate stage of orthodontics and combined with the orthodontic priority of the dentition, it can be observed that the premolars T24 and T25 move more obviously. Relying on the space vacated by them, the anterior dentition gradually moves towards the target posture as a whole. Defining the priority of orthodontics has a significant effect on the problem of orthodontic path planning, and it also verifies that the RRT algorithm after the improved extended node method can be applied to the problem of orthodontic path planning. This paper proposes to formulate a priority plan for orthodontics to find the breakthrough in orthodontics in advance and then use the RRT fast search random tree algorithm, which has the nature of full probability coverage. In combination, it can quickly and accurately find the solution close to the ideal orthodontic solution.

## 5. Conclusions

As a new type of orthodontic technology for orthodontics, bracketless correction technology has received extensive attention from researchers in the fields of computer-aided design and graphics in recent years. In the 1990s, some people have carried out research on invisible correction technology. After nearly 20 years of development, researchers have achieved a series of exploratory research results. Aiming at the problem of determining the ideal tooth pose, using Euler angles to define the tooth pose, an automatic tooth centering algorithm based on fitting and optimization is proposed. The position of each tooth in the 3D restoration was analyzed from a 2D perspective, and the weighted adjustment optimization method is used to calculate the displacement of the tooth coordinate and the rotation of the local coordinate axis to form related constraints, combined with the orthogonal bounding box collision detection method, developing an iterative algorithm based on the sudden descent method which is proposed to adjust the position of the teeth within the constraints of the spatial restoration curve. The automatic and final arrangement of the algorithm is done through experiments to improve the efficiency of the algorithm in order to compare the teeth with the existing methods of tooth alignment and to give the experimental results and analysis. At present, people's research on orthodontic technology without brackets is still in the laboratory stage. In practice, the core design process of invisible correction still adopts manual methods. Therefore, it is still a long time for the design process of invisible correction to be completed by computer. This article is divided into two parts to solve the problem of orthodontics. The first part is to determine the ideal orthodontic posture of the teeth. In this stage, the Euler angle is used to define the tooth posture, and the weighted fitting optimization method is used to calculate the displacement of the tooth coordinate and the rotation of the local coordinate axis to form the relevant constraint between the tooth posture and the prosthesis space curve, combined with the rectangular mark. The collision detection method of bounding box: the design is based on sudden descent method. However, compared with manual design, bracketless correction technology has the advantages of accuracy, speed, low cost, and high efficiency for automatic orthodontic design. Therefore, the bracketless correction technology has important theoretical research significance and practical value for orthodontic technology. It is worth investigating the problem of orthodontics without braces for researchers. Although this article has achieved certain results in solving the problem of digital orthodontics, these are only carried out in an experimental environment. In actual engineering applications, the situation is often more complicated and changeable, so there are still many shortcomings. In future research, we must also seek breakthroughs in these areas.

## Figures and Tables

**Table 1:** Correction time of transposition teeth.

Observed	15.3 ± 4.19	15.2 ± 3.15	38.5 ± 2.75
Matched	27.8 ± 3.28	29.4 ± 3.06	66.4 ± 3.67
*t*	14.254	16.559	24.115
*P*	*P* < 0.05	*P* < 0.05	*P* < 0.05

**Table 2:** Comparison of masticatory functions.

	Masticatory efficiency (%)	Occlusal force (lbs)
Observed	87.2% ± 5.6	154.6% ± 12.44
Matched	70.4% ± 6.7	105.3% ± 11.54
*T*	15.224	20.253
*P*	<0.05	<0.05

**Table 3:** Patient-specific treatment efficiency (n) of the two groups.

Group	Number	Valid	Inefficient	Total function
Observed	35	35	0	100.00%
Matched	35	33	2	94.29%
*P* value	—	0.05	<0.05	<0.05

**Table 4:** The beautiful, comfort, and convenient of two groups.

Group	Number	Aesthetics	Portable	Comfort
Observed	35	8.32 ± 2.21	9.13 ± 3.76	9.28 ± 3.41
Matched	35	6.57 ± 1.69	6.12 ± 2.45	6.03 ± 2.42
*t*-Value	—	4.893	7.886	5.645
*P* value	—	<0.05	<0.05	<0.05

## Data Availability

No data were used to support this study.
